# Low reticulocyte count at infusion is a risk factor for high-grade cytokine release syndrome in chimeric antigen receptor T cell therapy

**DOI:** 10.1007/s12185-025-04109-7

**Published:** 2025-11-17

**Authors:** Yusuke Tashiro, Tomoyasu Jo, Toshio Kitawaki, Noriyoshi Yoshinaga, Takashi Sakamoto, Kotaro Shirakawa, Junya Kanda, Momoko Nishikori, Kouhei Yamashita, Miki Nagao, Akifumi Takaori-Kondo, Yasuyuki Arai

**Affiliations:** 1 Department of Hematology, Graduate School of Medicine, Kyoto University, 54 Shogoin Kawahara-cho, Sakyo-ku, Kyoto, 606-8507 Japan; 2 Department of Cytotherapy, Kyoto University Hospital, 54 Shogoin Kawahara-cho, Sakyo-ku, Kyoto, 606-8507 Japan; 3 Center for Research and Application of Cellular Therapy, Kyoto University Hospital, 54 Shogoin Kawahara-cho, Sakyo-ku, Kyoto, 606-8507 Japan; 4 Department of Human Health Sciences, Graduate School of Medicine, Kyoto University, Kyoto, Japan

**Keywords:** Chimeric antigen receptor T-cell therapy, Cytokine release syndrome, Reticulocyte

## Abstract

Although chimeric antigen receptor (CAR)-T cell therapies are highly effective for B-cell lymphoma, they frequently cause cytokine release syndrome (CRS). High-grade CRS is serious and may require intensive care, yet reliable early predictive markers remain elusive. To identify risk factors for high-grade CRS, we retrospectively analyzed B-cell lymphoma patients who received CD19 CAR-T cell therapy. Of 106 patients analyzed, CRS occurred in 93 (88%), Grade ≥ 2 CRS in 28 (26%), and Grade ≥ 3 CRS in 6 (6%). Reticulocyte counts at infusion were significantly lower in patients who developed Grade ≥ 2 CRS (1.85 vs. 2.80 × 10^4^/µL, p = 0.02). Multivariate analysis identified low reticulocyte count (< 15,000/µL; HR 2.21; 95% CI 1.01–4.86; p = 0.048), high metabolic tumor volume (> 100 mL), and use of axicabtagene ciloleucel as independent risk factors for Grade ≥ 2 CRS. Stratification by the reticulocyte cutoff showed higher 30-day cumulative incidence of CRS in patients with low counts, for both Grade ≥ 2 (42.9% vs. 19.7%, p = 0.012) and Grade ≥ 3 CRS (17.9% vs. 1.3%, p < 0.001). KyoTox-CRS, a risk-scoring system integrating these factors, effectively stratified these CRS risks. Early prediction of high-grade CRS based on the reticulocyte count at infusion may help to guide optimal risk-based management of CAR-T cell therapy.

**Supplementary Information:**

The online version contains supplementary material available at 10.1007/s12185-025-04109-7.

## Introduction

Chimeric antigen receptor (CAR)-T cell therapies targeting CD19 have demonstrated significant efficacy in patients with relapsed or refractory B-cell lymphoma, as shown in both clinical trials [1–3], and real-world studies [4–7]. Their use as a promising treatment option has been expanding in clinical practice [8]. However, they are often accompanied by treatment-related toxicities, particularly cytokine release syndrome (CRS), infections and neurotoxic effects.

CRS is one of the most frequent and clinically significant acute-phase complications. In mild cases, it is usually limited to fever, headache, or fatigue, and may resolve with supportive care. However, in more severe cases, it can cause hypotension and hypoxia, sometimes leading to impaired consciousness and multi-organ dysfunction [9, 10]. CRS can cause not only reduced physical function, but also increased risk of immune effector cell-associated neurotoxicity syndrome (ICANS) and subsequent prolonged cytopenia [11, 12].

Despite its clinical significance, reliable markers that predict the early development of severe CRS have not been identified [13–15]. Cytokine panels including IL-6, IFN-γ, and IL-10 provide higher predictive accuracy [16, 17]. However, implementing routine real-time measurement of these cytokines is challenging in clinical practice. Furthermore, these panels are based on post-infusion changes and have limited value for early prediction. Having an early, reliable predictive marker would enable development of risk-adapted management strategies for each patient, ensuring safe administration of CAR-T cell therapy.

Thus, in this study, we aimed to identify early and reliable predictors of high-grade CRS by analyzing the association between laboratory and clinical parameters at the time of CAR-T cell infusion.

## Methods

### Patient population

This retrospective study enrolled consecutive adult patients with relapsed or refractory diffuse large B-cell lymphoma (DLBCL), or follicular lymphoma (FL) who underwent CD19-targeted CAR-T therapy, tisagenlecleucel (tisa-cel), lisocabtagene maraleucel (liso-cel), or axicabtagene ciloleucel (axi-cel), at Kyoto University Hospital between February 2019 and February 2024. All patients received lymphodepleting chemotherapy (fludarabine, cyclophosphamide, or bendamustine-based regimens) according to manufacturer instructions [2, 3, 18]. When CRS or ICANS was clinically suspected, corticosteroids and/or tocilizumab were administered at the discretion of the treating physicians, in accordance with the recommendations of the European Society for Blood and Marrow Transplantation (EBMT) and Joint Accreditation Committee ISCT-Europe & EBMT (JACIE) [19], and the official guidelines for the optimal use of each CAR-T product in Japan. Prophylactic administration of these agents was not performed in this cohort. Clinical and laboratory data were extracted from clinical records. This study was approved by the Ethics Committee of Kyoto University, and informed consent was obtained from all patients. All procedures were conducted in accordance with principles of the Declaration of Helsinki.

### Endpoints and definitions

The main outcome of interest in this study was the cumulative incidence of high-grade CRS, defined as Grade ≥ 2 CRS. This outcome was used to evaluate its association with multiple clinical and laboratory parameters at the time of CAR-T cell infusion. CRS and ICANS were diagnosed and graded according to criteria from the American Society for Transplantation and Cellular Therapy (ASTCT) [20]. We also evaluated CRS severity according to the modified CRS grading (m-CRS), in which Grade 1 was subdivided into Grade 1a (duration of fever ≤ 5 days) and Grade 1b (duration of fever > 5 days), as defined previously [12]. Laboratory parameters (white blood cell [WBC] count and differential, hemoglobin concentration, platelet count, reticulocyte count, LDH concentration, CRP concentration) were measured at infusion. The modified Endothelial Activation and Stress Index (mEASIX) score was calculated using LDH, CRP, and platelet count, as previously described [13]. Diagnoses of B-cell lymphoma, including DLBCL and FL, followed the WHO classification (5th edition) [21]. DLBCL cell-of-origin was determined by immunostaining (CD10, BCL6, and MUM1) according to the Hans classifier [22]. Disease status in DLBCL and FL patients at leukapheresis and infusion was assessed via fluorodeoxyglucose positron emission tomography/computed tomography (FDG-PET/CT), using the Revised Response Criteria for Malignant Lymphoma [23]. Metabolic tumor volume (MTV), evaluated based on FDG/PET, was calculated by defining tumor regions as areas with a standardized uptake value (SUV) of 2.5 or higher. Cutoff values for reticulocyte count and MTV to stratify Grade ≥ 2 CRS were determined using receiver operating characteristic (ROC) curve analysis.

### Statistical analysis

Continuous variables were compared using Student’s t-test, and categorical variables using Fisher's exact test. One-way ANOVA was used to analyze differences among three or more groups, followed by Tukey's multiple comparisons test for pairwise comparisons. Univariate and multivariate analyses for CRS, including cumulative incidence, were performed using Fine-Gray hazard models, considering death without CRS as a competing event. Variables with p < 0.05 in the univariate analysis were evaluated through stepwise inclusion and exclusion. The final model was selected based on the lowest value of the Bayesian Information Criterion (BIC). Adjusted hazard ratios (HRs) for cumulative incidence of Grade ≥ 2 CRS were converted to integer weights as follows: natural logarithmic HR values of < 0.50, and 0.50–1.00 were assigned respective weights of 0, and 1. The risk index was defined as the sum of these integer weights and used to stratify patients into risk groups. All statistical analyses were performed with EZR software (Jichi Medical University Saitama Medical Center) [24].

## Results

### Patient characteristics and laboratory data

The study cohort included 106 patients treated with tisa-cel (n = 76), liso-cel (n = 22), or axi-cel (n = 8) for relapsed or refractory DLBCL (n = 101) or FL (n = 5) (Table 1). The median age of patients at infusion was 63.5 years (range, 20–76). The median number of prior treatment lines was 4 (range, 2–11). Median reticulocyte count at the time of infusion was 2.57 × 10^4^/μL (institutional reference range: 2–10 × 10^4^/μL) (Table 2). At the time of infusion, 55% of patients were in complete response (CR) or partial response (PR), while 45% had stable disease (SD) or progressive disease (PD). Most patients (91%) received lymphodepleting chemotherapy with fludarabine and cyclophosphamide, while 6% with a bendamustine-based regimen. The median follow-up after CAR-T infusion was 398 days (range, 5–1970).

**Table 1:** Patient Characteristics

	Total	CRS ≥ Grade 2	CRS < Grade 2	p-value
	(N = 106)	(n = 28)	(n = 78)	
Gender, n				
Female	53 (50%)	13 (46%)	40 (51%)	0.83
Male	53 (50%)	15 (54%)	38 (49%)	
Age at infusion (years), median (range)	63.5 (20–73)	63.5 (20–73)	63.5 (27–76)	0.42
Disease, n				
FL	5 (5%)	1 (4%)	4 (5%)	1
DLBCL	101 (95%)	27 (96%)	74 (95%)	
Transformed (in DLBCL, n = 101), n				
Yes	24 (24%)	7 (26%)	17 (23%)	0.8
No	77 (76%)	20 (74%)	57 (77%)	
Hans (in DLBCL, n = 101), n				
GCB	49 (49%)	14 (52%)	35 (47%)	0.92
non-GCB	49 (49%)	12 (44%)	37 (50%)	
Missing	3 (3%)	1 (4%)	2 (3%)	
History of CNS involvement, n				
Yes	8 (8%)	4 (14%)	4 (5%)	0.2
No	98 (92%)	24 (86%)	74 (95%)	
Prior lines at infusion, n				
2–3	37 (35%)	13 (46%)	24 (31%)	0.17
> 3	69 (65%)	15 (54%)	54 (69%)	
Disease status at infusion, n				
CR/PR	58 (55%)	12 (43%)	46 (59%)	0.19
SD/PD	48 (45%)	16 (57%)	32 (41%)	
IPI (in DLBCL, n = 101) (at infusion), n				
Low	58 (57%)	12 (44%)	46 (62%)	0.15
Intermediate	17 (17%)	4 (15%)	13 (18%)	
High	24 (24%)	10 (37%)	14 (19%)	
MTV at infusion (mL), median (range)	10.7 (0–1332.9)	29.7 (0–1055.0)	6.7 (0–1332.9)	0.33
Lymphodepleting Regimen, n				
Fly/Cy	97 (91%)	24 (89%)	73 (94%)	0.03*
Bendamustine based	6 (6%)	1 (4%)	5 (6%)	
others	3 (3%)	3 (7%)	0 (0%)	
CAR-T, n				
tisa-cel	76 (72%)	22 (79%)	54 (69%)	< 0.01*
liso-cel	22 (21%)	1 (4%)	21 (27%)	
axi-cel	8 (8%)	5 (18%)	3 (4%)	
CRS, n				
No	13 (12%)	0 (0%)	13 (17%)	
Grade 1a	27 (25%)	0 (0%)	27 (35%)	
Grade 1b	38 (36%)	0 (0%)	38 (49%)	
Grade 2	22 (21%)	22 (79%)	0 (0%)	
Grade 3	2 (2%)	2 (7%)	0 (0%)	
Grade 4	4 (4%)	4 (14%)	0 (0%)	
ICANS, n				
Yes	17 (16%)	11 (39%)	6 (8%)	< 0.01*
No	89 (84%)	17 (61%)	72 (92%)	
Tocilizumab administration				
Yes	73 (69%)	23 (82%)	50 (64%)	0.10
No	33 (31%)	5 (18%)	28 (36%)	
Corticosteroid administration				
Yes	32 (30%)	16 (57%)	16 (21%)	< 0.01*
No	74 (70%)	12 (43%)	62 (79%)	
Best response, n				
CR/PR	85 (80%)	22 (79%)	63 (81%)	0.79
SD/PD	21 (20%)	6 (21%)	15 (19%)	

*axi-cel* axicabtagene ciloleucel, *CAR-T* chimeric antigen receptor-T cell, *CNS* central nervous system, *CR* complete response, *CRS* cytokine release syndrome, *DLBCL* diffuse large B-cell lymphoma, *Flu/Cy* fludarabine and cyclophosphamide, *FL* follicular lymphoma, *GCB* germinal center B-cell–like, *ICANS* immune effector cell-associated neurotoxicity syndrome, *IPI* international prognostic index, *liso-cel* lisocabtagene maraleucel, *MTV* metabolic tumor volume, *PD* progressive disease, *PR* partial response, *SD* stable disease, *tisa-cel* tisagenlecleucel

Bolded p-values and those marked with an asterisk *indicates p < 0.05

**Table 2:** Laboratory Data at Infusion

Day0	Total	CRS ≥ Grade2	CRS < Grade2	p-value
	(N = 106)	(n = 28)	(n = 78)	
Reticulocyte (10^4^/μL)	2.57 (0.36–6.75)	1.85 (0.37–5.26)	2.80 (0.36–6.75)	0.02*
WBC (10^9^/L)	1.49 (0–7.43)	1.00 (0–5.57)	1.72 (0.07–7.43)	0.04*
Neut (%)	87.7 (11.9–98.0)	87.3 (17.3–96.0)	88.0 (11.9–98.0)	0.55
Lymph (%)	2.0 (0–73.5)	2.4 (0–19.0)	2.0 (0–73.5)	0.66
Mono (%)	4.5 (0–30.0)	3.2 (0–30.0)	5.0 (0–28.0)	0.72
Eosino (%)	3.4 (0–56.9)	3.2 (0–56.9)	3.6 (0–34.8)	0.90
Baso (%)	0 (0–4.8)	0 (0–1.7)	0 (0–4.8)	0.74
Plt (× 10^4^/μL)	13.2 (1.0–32.7)	12.3 (1.0–27.8)	13.9 (1.8–32.7)	0.42
LDH (U/L)	215 (146–2400)	237 (157–2400)	214 (146–1066)	0.04*
CRP (mg/dL)	0.45 (0–19.12)	1.00 (0.10–19.12)	0.40 (0–11.90)	0.02*
mEASIX score	0.82 (0–4588.80)	1.54 (1.22–4588.80)	0.57 (0–667.65)	0.09

Laboratory data at Day 0 are shown as median (range)

*Baso* basophils, *Eosino* eosinophils, *Lymph* lymphocytes, *mEASIX* modified Endothelial Activation and Stress Index, *Mono* monocytes, *Neut* neutrophils, *Plt* platelets, *WBC* white blood cells

Other abbreviations are shown in Table 1

Bolded p-values and those marked with an asterisk *indicates p < 0.05

**Fig. 1:**
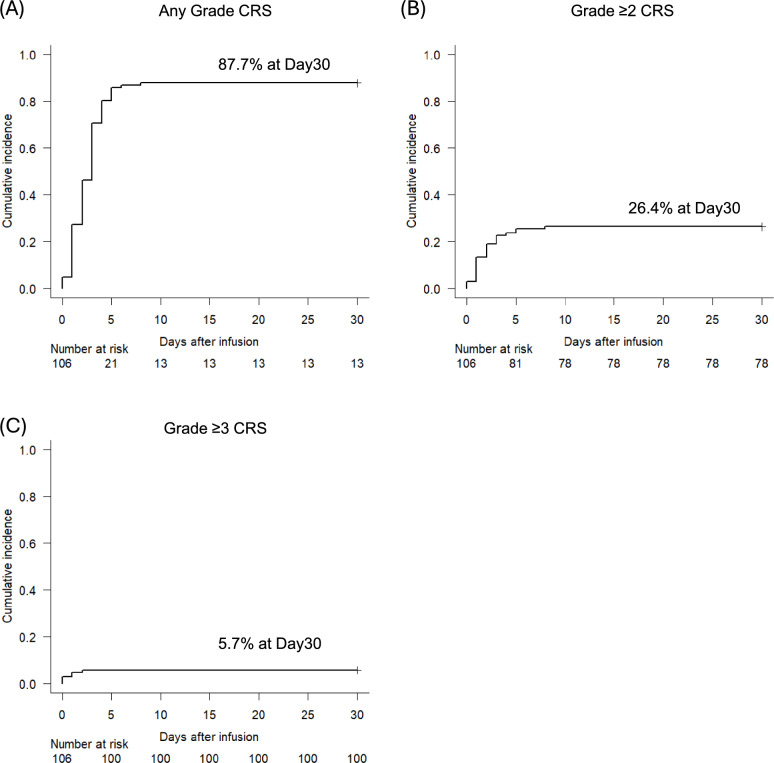
Cumulative incidence of cytokine release syndrome (CRS) in patients with lymphoma receiving chimeric antigen receptor (CAR)-T cell therapy. **A** Any grade CRS. 87.7% at 30-day post-infusion. **B** Grade ≥ 2 CRS. 26.4% at 30-day post-infusion. **C** Grade ≥ 3 CRS. 5.7% at 30-day post-infusion

CRS occurred in 93 patients (88%), with a median onset of 2 days after CAR-T infusion. Grade ≥ 2 and Grade ≥ 3 CRS occurred in 28 (26%) and 6 (6%) patients (Fig. 1). Severity was distributed as Grade 1a in 27 patients, Grade1b in 38, Grade 2 in 22, Grade 3 in 2, and Grade 4 in 4 patients. By CAR-T product, CRS incidence was 86% with tisa-cel, 90% with liso-cel, and 100% with axi-cel. For Grade ≥ 2 CRS, the incidence was 29% with tisa-cel, 5% with liso-cel, and 50% with axi-cel (Table 3). Two patients died from disease progression within 30 days after infusion, on days 5 and 6, respectively. Tocilizumab and corticosteroids were administered in 82%, and 57% of patients who developed Grade ≥ 2 CRS, respectively, for management of either CRS or ICANS.

**Table 3:** Frequency of CRS by Grade for Each CAR-T Product

CRS Grade	Total	Tisa-cel	Liso-cel	Axi-cel
	(N = 106)	(n = 76)	(n = 22)	(n = 8)
Grade 0 (No CRS)	13 (12%)	11 (14%)	2 (9%)	0 (0%)
Any CRS (Grade ≥ 1)	93 (88%)	65 (86%)	20 (90%)	8 (100%)
Grade ≥ 2 CRS	27 (25%)	22 (29%)	1 (5%)	4 (50%)
Grade ≥ 3 CRS	6 (6%)	5 (7%)	0 (0%)	1 (13%)
Grade 1a	27 (25%)	16 (21%)	9 (41%)	2 (50%)
Grade 1b	39 (37%)	27 (36%)	10 (45%)	2 (50%)
Grade 2	21 (20%)	17 (22%)	1 (5%)	3 (37%)
Grade 3	2 (2%)	2 (3%)	0 (0%)	0 (0%)
Grade 4	4 (4%)	3 (4%)	0 (0%)	1 (13%)

Abbreviations are shown in Table 1

### Comparison of patient characteristics between patients with and without Grade ≥ 2 CRS

We compared patient characteristics and laboratory data between those who developed Grade ≥ 2 CRS and those who did not (Tables 1 and 2). The proportion of axi-cel use was significantly higher among patients with Grade ≥ 2 CRS, compared to those without. There were no significant differences in sex, age, disease subtype, or number of prior treatment lines at infusion (Table 1). Regarding laboratory data, reticulocyte counts were significantly lower in patients with Grade ≥ 2 CRS compared to those without (1.85 vs. 2.80 × 10^4^/μL, *p* = 0.02). Similarly, WBC counts were significantly lower in patients with Grade ≥ 2 CRS compared to those without (1.00 vs 1.72 × 10^9^/L, *p* = 0.04) (Table 2). In addition, LDH (237 vs 214 U/L, *p* = 0.04) and CRP (1.00 vs 0.40 mg/dL, *p* = 0.02), were significantly higher in patients who developed Grade ≥ 2 CRS. No other individual markers, including platelet count, showed significant differences between the two groups. Patients with Grade ≥ 2 CRS more frequently developed ICANS, and required corticosteroid administration.

### Univariate analysis for development of Grade ≥ 2 CRS

To identify potential risk factors associated with Grade ≥ 2 CRS development, we first performed univariate Fine-Gray hazard analysis. Based on ROC curve analysis, cutoff values to stratify Grade ≥ 2 were 15,000/µL for reticulocyte count, and 100 mL for MTV (Figure S1). High IPI score, MTV at infusion, and the use of axi-cel were significantly associated with increased cumulative incidence of Grade ≥ 2 CRS. Among laboratory parameters, lower reticulocyte and WBC counts, higher LDH and CRP levels were also significantly associated with Grade ≥ 2 CRS (Supplemental Table 1). There was no significant association between Grade ≥ 2 CRS development and platelet count.

### Multivariate analysis for development of Grade ≥ 2 CRS

**Fig. 2:**
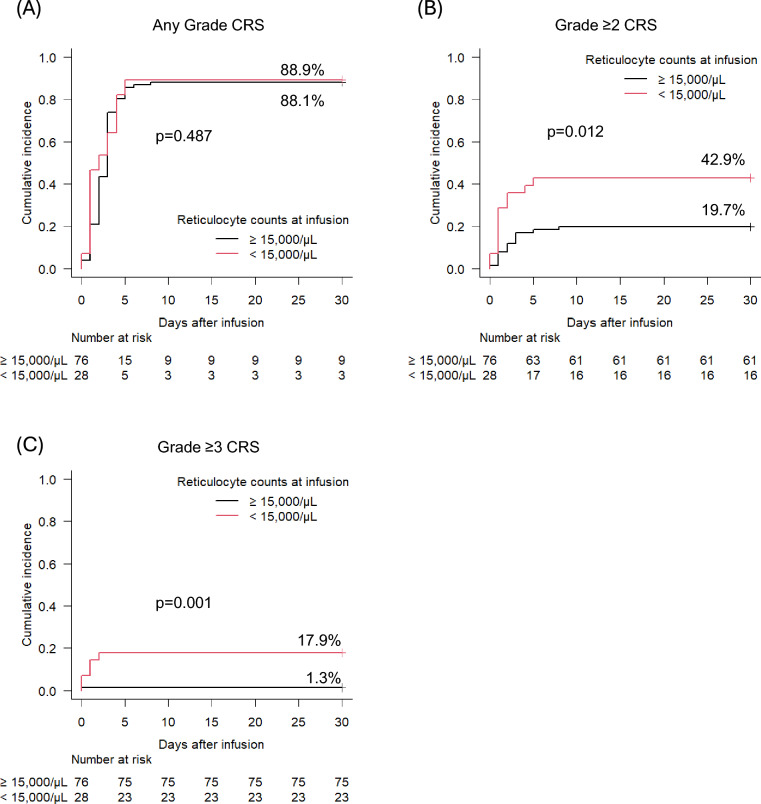
Cumulative incidence of cytokine release syndrome (CRS) stratified by reticulocyte counts. **A** Any grade CRS. **B** Grade ≥ 2 CRS. **C** Grade ≥ 3 CRS

Factors that were significant in the univariate analysis were included for multivariate analysis, as described in the Methods. Multivariate analysis identified low reticulocyte count (< 15,000/µL; HR 2.21; 95% CI, 1.01–4.86; p = 0.048), high MTV (> 100 mL; HR 2.63; 95% CI, 1.22–5.71; p = 0.014) and use of axi-cel (HR 2.64; 95% CI, 1.15–6.07; p = 0.023) as independent risk factors for development of Grade ≥ 2 CRS (Table 4). Patients with lower reticulocyte counts were more likely to develop higher-grade CRS (Supplementary Table 2, and Supplementary Fig. 2). When patients were stratified by reticulocyte count according to the ROC-derived cutoff, those with low counts had a significantly higher cumulative incidence of Grade ≥ 2 CRS (42.9% vs. 19.7%, p = 0.012) and Grade ≥ 3 CRS (17.9% vs. 1.3%, p < 0.001), whereas there was no significant difference in cumulative incidence of CRS for any grade, including low-grade CRS (88.9% vs. 88.1%) (Fig. 2A–C).

**Table 4:** Multivariate Analysis for Grade ≥ 2 CRS

	HR (95% CI)	p-value
Reticulocyte		
≥ 15,000/μL	1	
< 15,000/μL	2.21 (1.01—4.86)	0.048*
MTV		
< 100 mL	1	
≥ 100 mL	2.63 (1.22—5.71)	0.014*
CAR-T product		
tisa-cel or liso-cel	1	
axi-cel	2.64 (1.15—6.07)	0.023*

*CI* confidence interval, *HR* hazard ratio

Other abbreviations are shown in Table 1

Bolded p-values and those marked with an asterisk *indicates p < 0.05

### Development of a risk score for Grade ≥ 2 CRS development: Kyoto University-developed CRS prediction model using a reticulocyte-based scoring system (KyoTox-CRS)

To weight the impact of each clinical factor, a risk scoring system for use in clinical settings was developed. Based on the multivariate analysis, we defined a Kyoto University-developed CRS prediction model using a reticulocyte-based scoring system (KyoTox-CRS), including three variables with independent impact on Grade ≥ 2 CRS: reticulocyte count at infusion, MTV at infusion, and CAR-T product. One point was assigned for reticulocyte count < 15,000/µL, MTV > 100 mL, and axi-cel (Supplemental Table 3). The KyoTox-CRS score was determined by summing these three factors, and patients were divided into the following three risk groups: low risk (score 0), intermediate risk (score 1), and high risk (score 2–3).

**Fig. 3:**
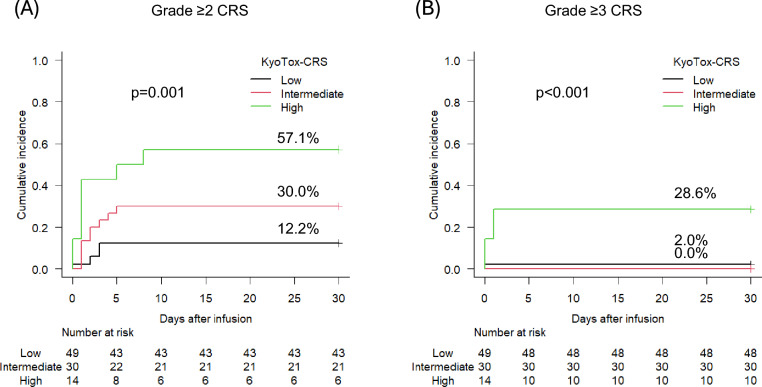
Cumulative incidence of cytokine release syndrome (CRS) stratified by KyoTox-CRS. **A** Grade ≥ 2 CRS. **B** Grade ≥ 3 CRS. Among the total study population (n = 106), 93 patients with complete data for reticulocyte count, MTV, and axi-cel use were included in this analysis. Thirteen patients were excluded due to missing MTV or reticulocyte count data

KyoTox-CRS effectively stratified the likelihood of developing Grade ≥ 2 CRS (57.1% in the high-risk group, 30.0% in the intermediate-risk group, and 12.2% in the low-risk group, p = 0.001), and identified patients with high risk for Grade ≥ 3 CRS (28.6% in the high-risk group, 0.0% in the intermediate-risk group, and 2.0% in the low-risk group, p < 0.001) (Fig. 3, Supplemental Table 4, and Supplemental Fig. 3).

## Discussion

By evaluating the influence of various clinical and hematologic parameters on development of high-grade CRS, we first identified low reticulocyte count at the time of CAR-T infusion as a novel risk factor for high-grade CRS development. In addition, a novel risk stratification system, KyoTox-CRS, incorporating a low reticulocyte count, high MTV and axi-cel treatment, was developed to predict incidence of high-grade CRS after CAR-T cell therapy in patients with relapsed or refractory B-cell lymphoma. This is the first report to demonstrate an association between low reticulocyte count and high-grade CRS.

Although management strategies for CRS have improved, it remains a serious complication in the real world. Further efforts are needed to create precise risk prediction models. In our study, CRS of any grade occurred in 88% of patients, with a median onset of day 2 (range 0–8). Grade ≥ 2 and ≥ 3 CRS occurred in 26% and 6% of patients, respectively. These results are consistent with previous reports [1, 3, 13, 25]. Since CRS developed shortly after infusion in our cohort, markers that can predict its development as early as the time of infusion are required.

Our multivariate analysis identified low reticulocyte count at infusion as an independent risk factor for developing Grade ≥ 2 CRS. In our study, patients had received a median of four prior lines of therapy. Previous studies have indicated that reticulocyte count can serve as a marker of bone marrow suppression induced by cytotoxic chemotherapy [26, 27]. Furthermore, a report has suggested that disease status necessitating repeated chemotherapy is associated with an increased risk of CRS [28]. These observations imply that a reduced reticulocyte count may provide a simple and informative surrogate marker of cumulative treatment-related hematopoietic toxicity, which may be associated with poor bone marrow reserve and, in turn, predispose patients to the development of high-grade CRS.

While low WBC counts, another hematological parameter, were also associated with Grade ≥ 2 CRS in the univariate analysis, this association was not significant in the multivariate analysis. Among different combinations of candidate variables, reticulocyte counts consistently demonstrated greater predictive value than WBC counts. Advanced CRS occurs in the presence of elevated inflammatory cytokines, such as IL-6, which can suppress hematopoiesis and reduce production of both reticulocytes and WBCs. Reticulocytes may be particularly vulnerable because inflammatory cytokines also impair iron utilization by increasing hepcidin production [29, 30], and by activating pro-inflammatory M1 macrophages [31]. Indeed, consistent with previous studies [13, 32], in our univariate analysis, elevated CRP at the time of CAR-T cell infusion, an inflammatory marker, was associated with an increased risk of Grade ≥ 2 CRS. While, in our multivariate analysis, CRP did not remain a significant predictor, possibly due to the collinearity between CRP and reticulocyte count and the limited sample size, these results suggest that systemic inflammation may contribute to CRS development. Thus, reduced erythropoiesis, reflected in a low reticulocyte count, may serve as a marker both of cumulative hematopoietic toxicity from prior therapies, and of an inflammatory milieu that facilitates development of high-grade CRS [33]. In line with this, higher red cell distribution width (RDW), a marker of disrupted erythropoiesis, is reportedly associated with systemic inflammation and poor outcomes following CAR-T cell therapy [34].

Our analysis identified high MTV as a risk factor for Grade ≥ 2 CRS, consistent with previous reports showing that high tumor burden before CAR-T infusion is a key risk factor for Grade ≥ 3 CRS [35–37]. In our study, the ROC-derived cutoff value for MTV that represented an increased likelihood of Grade ≥ 2 CRS was 100 mL. Previous studies have reported cutoff values ranging from 26.37 to 147.5 mL for predicting Grade ≥ 3 CRS [36, 37]. Such variation may reflect differences in patient backgrounds and institutional variability in diagnostic and therapeutic practices. Our study emphasizes the importance of paying particular attention to high-grade CRS when tumor volume exceeds these levels.

In this study, Grade ≥ 2 CRS occurred in 29% of patients receiving tisa-cel, 5% with liso-cel, and 50% with axi-cel. Grade ≥ 3 CRS occurred in 7% of patients receiving tisa-cel, 0% with liso-cel, and 13% with axi-cel. These findings are generally similar to previous reports, which documented Grade ≥ 3 CRS in 6%, 3%, and 9% of patients receiving tisa-cel, liso-cel, and axi-cel, respectively [7, 25, 38]. In our multivariate analysis, axi-cel was significantly associated with a higher risk of Grade ≥ 2 CRS. This might be attributed to the CD28 costimulatory domain of axi-cel, which promotes rapid CAR-T cell expansion and early, high-level cytokine release [39]. Although our analysis is based on a limited number of patients, which may affect the robustness of the findings, the observed trend is consistent with previous studies reporting a higher risk of CRS with axi-cel compared to tisa-cel or liso-cel [6, 40] These results may indicate that patients receiving axi-cel should be carefully monitored for the potential development of high-grade CRS.

Based on results of the multivariate analysis, we developed KyoTox-CRS, a scoring system that predicts Grade ≥ 2 CRS. In this system, one point is assigned for each of the following factors: reticulocyte < 15,000/µL, MTV > 100 mL, and use of axi-cel. KyoTox-CRS stratified patients into three distinct groups for Grade ≥ 2 CRS (high-, intermediate-, and low-risk groups). Moreover, KyoTox-CRS can be used to predict development of Grade ≥ 3 CRS, as 28.6% among KyoTox-CRS high-risk group, with significant risk enhancement. These results suggest that our risk score could be used to identify patients who would benefit from more proactive tocilizumab and corticosteroid treatment to prevent CRS progression.

This study has several limitations. First, it was a retrospective, single-center study, which may have introduced biases related to patient selection and institutional practices. Second, only eight patients in our cohort received axi-cel therapy. This small number may have limited the statistical power to accurately evaluate the risk of CRS with axi-cel. Third, validation using data from other institutions regarding the significance of reticulocyte counts and KyoTox-CRS has not been performed. Since reticulocyte counts have not previously been a focus of attention, it is difficult to verify them using existing data. To validate our findings, further studies with larger cohorts and attention to reticulocyte data are warranted. Fourth, the management of CRS has evolved over time, with a trend toward earlier use of tocilizumab and corticosteroids in recent years [41]. Given the retrospective nature of this study, potential temporal changes in CRS management may have influenced our results.

In conclusion, we identified low reticulocyte count at infusion as a novel and practical biomarker for predicting high-grade CRS. Building on this finding, we developed KyoTox-CRS, a simple risk score that incorporates reticulocyte count, MTV, and type of CAR-T product. This scoring system may allow early identification of patients at high risk for severe CRS, enabling timely intervention with tocilizumab or corticosteroids, to improve prediction, clinical management, and optimization of CAR-T therapy.

## Supplementary Information

Below is the link to the electronic supplementary material.

Supplementary file1 (PDF 292 KB)

## Data Availability

Data that support findings of this study are available from the corresponding author upon request.

## Declarations

### Data sharing statement

Data employed in this study are not publicly available due to ethical restrictions. Releasing these data would exceed the scope of patient consent for research use.

## References

[1] Neelapu SS, Locke FL, Bartlett NL, Lekakis LJ, Miklos DB, Jacobson CA, et al. Axicabtagene ciloleucel CAR T-cell therapy in refractory large B-cell lymphoma. N Engl J Med. 2017;377(26):2531–44. 29226797 10.1056/NEJMoa1707447 PMC5882485

[2] Schuster SJ, Bishop MR, Tam CS, Waller EK, Borchmann P, McGuirk JP, et al. Tisagenlecleucel in adult relapsed or refractory diffuse large B-cell lymphoma. N Engl J Med. 2019;380(1):45–56. 30501490 10.1056/NEJMoa1804980

[3] Abramson JS, Palomba ML, Gordon LI, Lunning MA, Wang M, Arnason J, et al. Lisocabtagene maraleucel for patients with relapsed or refractory large B-cell lymphomas (TRANSCEND NHL 001): a multicentre seamless design study. Lancet. 2020;396(10254):839–52. 10.1016/S0140-6736(20)31366-0. 32888407 10.1016/S0140-6736(20)31366-0

[4] Nastoupil LJ, Jain MD, Feng L, Spiegel JY, Ghobadi A, Lin Y, et al. Standard-of-care axicabtagene ciloleucel for relapsed or refractory large B-cell lymphoma: results from the US lymphoma CAR T consortium. J Clin Oncol. 2020;38(27):3119–28. 32401634 10.1200/JCO.19.02104 PMC7499611

[5] Pasquini MC, Hu ZH, Curran K, Laetsch T, Locke F, Rouce R, et al. Real-world evidence of tisagenlecleucel for pediatric acute lymphoblastic leukemia and non-Hodgkin lymphoma. Blood Adv. 2020;4(21):5414–24. 10.1182/bloodadvances.2020003092. 33147337 10.1182/bloodadvances.2020003092 PMC7656920

[6] Bachy E, Le Gouill S, Di Blasi R, Sesques P, Manson G, Cartron G, et al. A real-world comparison of tisagenlecleucel and axicabtagene ciloleucel CAR T cells in relapsed or refractory diffuse large B cell lymphoma. Nat Med. 2022;28(10):2145–54. 36138152 10.1038/s41591-022-01969-y PMC9556323

[7] Riedell PA, Grady CB, Nastoupil LJ, Luna A, Ahmed N, Richard TM, et al. Lisocabtagene maraleucel for relapsed/refractory large B-cell lymphoma: a cell therapy consortium real-world analysis. Blood Adv. 2025;9(5):1232–41. 39657136 10.1182/bloodadvances.2024014164 PMC11993828

[8] Goto H, Onozawa M, Teshima T. Novel CAR T cell therapies for patients with large B cell lymphoma. Int J Hematol. 2024;120(1):6–14. 38795249 10.1007/s12185-024-03792-2

[9] Neelapu SS, Tummala S, Kebriaei P, Wierda W, Gutierrez C, Locke FL, et al. Chimeric antigen receptor T-cell therapy - assessment and management of toxicities. Nat Rev Clin Oncol. 2018;15(1):47–62. 28925994 10.1038/nrclinonc.2017.148 PMC6733403

[10] Lee DW, Gardner R, Porter DL, Louis CU, Ahmed N, Jensen M, et al. Current concepts in the diagnosis and management of cytokine release syndrome. Blood. 2014;124(2):188–95. 24876563 10.1182/blood-2014-05-552729 PMC4093680

[11] Hamada R, Arai Y, Kitawaki T, Nakamura N, Murao M, Matsushita M, et al. Fluctuation of physical function during chimeric antigen receptor T-cell therapy during rehabilitation intervention: real-world data and risk factor analyses. EJHaem. 2024;5(6):1252–9. 39691237 10.1002/jha2.1043 PMC11647737

[12] Nakamura N, Jo T, Arai Y, Kitawaki T, Nishikori M, Mizumoto C, et al. Clinical impact of cytokine release syndrome on prolonged hematotoxicity after chimeric antigen receptor T cell therapy: KyoTox A-Score, a novel prediction model. Transplant Cell Ther. 2024;30(4):404–14. 10.1016/j.jtct.2024.01.073. 38281589 10.1016/j.jtct.2024.01.073

[13] Pennisi M, Sanchez-Escamilla M, Flynn JR, Shouval R, Tomas AA, Silverberg ML, et al. Modified EASIX predicts severe cytokine release syndrome and neurotoxicity after chimeric antigen receptor T cells. Blood Adv. 2021;5(17):3397–406. 10.1182/bloodadvances.2020003885. 34432870 10.1182/bloodadvances.2020003885 PMC8525234

[14] Sesques P, Kirkwood AA, Kwon M, Rejeski K, Jain MD, Di Blasi R, et al. Novel prognostic scoring systems for severe CRS and ICANS after anti-CD19 CAR T cells in large B-cell lymphoma. J Hematol Oncol. 2024;17(1):61. 39107847 10.1186/s13045-024-01579-w PMC11305039

[15] Wakabayashi H, Terakura S, Ishigiwa K, Ohara F, Hirano S, Yokota H, et al. Simple and early prediction of severe CAR-T-related adverse events after Axi-cel infusion by initial high fever. Int J Hematol. 2025;122(1):106–16. 40014276 10.1007/s12185-025-03957-7

[16] Teachey DT, Lacey SF, Shaw PA, Melenhorst JJ, Maude SL, Frey N, et al. Identification of predictive biomarkers for cytokine release syndrome after chimeric antigen receptor T-cell therapy for acute lymphoblastic leukemia. Cancer Discov. 2016;6(6):664–79. 27076371 10.1158/2159-8290.CD-16-0040 PMC5448406

[17] Su M, Chen L, Xie L, Fleurie A, Jonquieres R, Cao Q, et al. Identification of early predictive biomarkers for severe cytokine release syndrome in pediatric patients with chimeric antigen receptor T-cell therapy. Front Immunol. 2024;15:1450173. 39328408 10.3389/fimmu.2024.1450173 PMC11424402

[18] Locke FL, Ghobadi A, Jacobson CA, Miklos DB, Lekakis LJ, Oluwole OO, et al. Long-term safety and activity of axicabtagene ciloleucel in refractory large B-cell lymphoma (ZUMA-1): a single-arm, multicentre, phase 1-2 trial. Lancet Oncol. 2019;20(1):31–42. 10.1016/S1470-2045(18)30864-7. 30518502 10.1016/S1470-2045(18)30864-7 PMC6733402

[19] Yakoub-Agha I, Chabannon C, Bader P, Basak GW, Bonig H, Ciceri F, et al. Management of adults and children undergoing chimeric antigen receptor T-cell therapy: best practice recommendations of the European Society for Blood and Marrow Transplantation (EBMT) and the Joint Accreditation Committee of ISCT and EBMT (JACIE). Haematologica. 2020;105(2):297–316. 31753925 10.3324/haematol.2019.229781 PMC7012497

[20] Lee DW, Santomasso BD, Locke FL, Ghobadi A, Turtle CJ, Brudno JN, et al. ASTCT consensus grading for cytokine release syndrome and neurologic toxicity associated with immune effector cells. Biol Blood Marrow Transplant. 2019;25(4):625–38. 10.1016/j.bbmt.2018.12.758. 30592986 10.1016/j.bbmt.2018.12.758 PMC12180426

[21] Alaggio R, Amador C, Anagnostopoulos I, Attygalle AD, Araujo IBO, Berti E, et al. The 5th edition of the World Health Organization classification of haematolymphoid tumours: lymphoid neoplasms. Leukemia. 2022;36(7):1720–48. 35732829 10.1038/s41375-022-01620-2 PMC9214472

[22] Hans CP, Weisenburger DD, Greiner TC, Gascoyne RD, Delabie J, Ott G, et al. Confirmation of the molecular classification of diffuse large B-cell lymphoma by immunohistochemistry using a tissue microarray. Blood. 2004;103(1):275–82. 14504078 10.1182/blood-2003-05-1545

[23] Cheson BD, Pfistner B, Juweid ME, Gascoyne RD, Specht L, Horning SJ, et al. Revised response criteria for malignant lymphoma. J Clin Oncol. 2007;25(5):579–86. 17242396 10.1200/JCO.2006.09.2403

[24] Kanda Y. Investigation of the freely available easy-to-use software “EZR” for medical statistics. Bone Marrow Transplant. 2013;48(3):452–8. 23208313 10.1038/bmt.2012.244 PMC3590441

[25] Landsburg DJ, Frigault MJ, Heim M, Foley SR, Hill B, Schofield G, et al. Real-world outcomes with tisagenlecleucel in aggressive B-cell lymphoma: subgroup analyses from the CIBMTR registry. J Immunother Cancer. 2025;13(2):e009890. 10.1136/jitc-2024-009890. 39924174 10.1136/jitc-2024-009890 PMC11808862

[26] Torres A, Sánchez J, Lakomsky D, Serrano J, Alvarez MA, Martín C, et al. Assessment of hematologic progenitor engraftment by complete reticulocyte maturation parameters after autologous and allogeneic hematopoietic stem cell transplantation. Haematologica. 2001;86(1):24–9. 11146566

[27] Gonçalo AP, Barbosa IL, Campilho F, Campos A, Mendes C. Predictive value of immature reticulocyte and platelet fractions in hematopoietic recovery of allograft patients. Transplant Proc. 2011;43(1):241–3. 10.1016/j.transproceed.2010.12.030. 21335197 10.1016/j.transproceed.2010.12.030

[28] Mueller KT, Maude SL, Porter DL, Frey N, Wood P, Han X, et al. Cellular kinetics of CTL019 in relapsed/refractory B-cell acute lymphoblastic leukemia and chronic lymphocytic leukemia. Blood. 2017;130(21):2317–25. 28935694 10.1182/blood-2017-06-786129 PMC5731220

[29] Si X, Gu T, Liu L, Huang Y, Han Y, Qian P, et al. Hematologic cytopenia post CAR T cell therapy: etiology, potential mechanisms and perspective. Cancer Lett. 2022;550:215920. 10.1016/j.canlet.2022.215920. 36122628 10.1016/j.canlet.2022.215920

[30] Read JA, Rouce RH, Mo F, Mamonkin M, King KY. Apoptosis of hematopoietic stem cells contributes to bone marrow suppression following chimeric antigen receptor T cell therapy. Transplant Cell Ther. 2023;29(3):165.e1-165.e7. 10.1016/j.jtct.2022.12.020. 36592718 10.1016/j.jtct.2022.12.020 PMC9991966

[31] Xiao X, Huang S, Chen S, Wang Y, Sun Q, Xu X, et al. Mechanisms of cytokine release syndrome and neurotoxicity of CAR T-cell therapy and associated prevention and management strategies. J Exp Clin Cancer Res. 2021;40(1):367. 34794490 10.1186/s13046-021-02148-6 PMC8600921

[32] Greenbaum U, Strati P, Saliba RM, Torres J, Rondon G, Nieto Y, et al. CRP and ferritin in addition to the EASIX score predict CAR-T-related toxicity. Blood Adv. 2021;5(14):2799–806. 10.1182/bloodadvances.2021004575. 34264268 10.1182/bloodadvances.2021004575 PMC8341350

[33] Pérez S, Rius-Pérez S. Macrophage polarization and reprogramming in acute inflammation: a redox perspective. Antioxidants. 2022;11(7):1394. 10.3390/antiox11071394. 35883885 10.3390/antiox11071394 PMC9311967

[34] Nakamura N, Jo T, Arai Y, Kitawaki T, Nishikori M, Mizumoto C, et al. Utilizing red blood cell distribution width (RDW) as a reliable biomarker to predict treatment effects after chimeric antigen receptor T cell therapy. Clin Exp Med. 2024;24(1):105. 38771501 10.1007/s10238-024-01373-5 PMC11108946

[35] Shimabukuro-Vornhagen A, Gödel P, Subklewe M, Stemmler HJ, Schlößer HA, Schlaak M, et al. Cytokine release syndrome. J Immunother Cancer. 2018;6(1):56. 10.1186/s40425-018-0343-9. 29907163 10.1186/s40425-018-0343-9 PMC6003181

[36] Hong R, Tan Su Yin E, Wang L, Zhao X, Zhou L, Wang G, et al. Tumor burden measured by 18F-FDG PET/CT in predicting efficacy and adverse effects of chimeric antigen receptor T-cell therapy in non-Hodgkin lymphoma. Front Oncol. 2021;11:713577. 34422666 10.3389/fonc.2021.713577 PMC8371710

[37] Dean EA, Mhaskar RS, Lu H, Mousa MS, Krivenko GS, Lazaryan A, et al. High metabolic tumor volume is associated with decreased efficacy of axicabtagene ciloleucel in large B-cell lymphoma. Blood Adv. 2020;4(14):3268–76. 32702097 10.1182/bloodadvances.2020001900 PMC7391155

[38] Strati P, Nastoupil LJ, Westin J, Fayad LE, Ahmed S, Fowler NH, et al. Clinical and radiologic correlates of neurotoxicity after axicabtagene ciloleucel in large B-cell lymphoma. Blood Adv. 2020;4(16):3943–51. 10.1182/bloodadvances.2020002228. 32822484 10.1182/bloodadvances.2020002228 PMC7448589

[39] Morris EC, Neelapu SS, Giavridis T, Sadelain M. Cytokine release syndrome and associated neurotoxicity in cancer immunotherapy. Nat Rev Immunol. 2022;22(2):85–96. 34002066 10.1038/s41577-021-00547-6 PMC8127450

[40] Looka A, Qualls DA, Matthews D, Redd RA, Sakellis C, Duffy C, et al. A real-world comparison of commercial-use axicabtagene ciloleucel and lisocabtagene maraleucel in large B-cell lymphoma. Blood Adv. 2025;9(3):455–62. 10.1182/bloodadvances.2024012992. 39546746 10.1182/bloodadvances.2024012992 PMC11808612

[41] Jain MD, Smith M, Shah NN. How I treat refractory CRS and ICANS after CAR T-cell therapy. Blood. 2023;141(20):2430–42. 10.1182/blood.2022017414. 36989488 10.1182/blood.2022017414 PMC10329191

