# Correction to: Low reticulocyte count at infusion is a risk factor for high-grade cytokine release syndrome in chimeric antigen receptor T cell therapy

**DOI:** 10.1007/s12185-026-04235-w

**Published:** 2026-06-25

**Authors:** Yusuke Tashiro, Tomoyasu Jo, Toshio Kitawaki, Noriyoshi Yoshinaga, Takashi Sakamoto, Kotaro Shirakawa, Junya Kanda, Momoko Nishikori, Kouhei Yamashita, Miki Nagao, Akifumi Takaori-Kondo, Yasuyuki Arai

**Affiliations:** 1 Department of Hematology, Graduate School of Medicine, Kyoto University, 54 Shogoin Kawahara-Cho, Sakyo-Ku, Kyoto, 606-8507 Japan; 2 Department of Cytotherapy, Kyoto University Hospital, 54 Shogoin Kawahara-Cho, Sakyo-Ku, Kyoto, 606-8507 Japan; 3 Center for Research and Application of Cellular Therapy, Kyoto University Hospital, 54 Shogoin Kawahara-Cho, Sakyo-Ku, Kyoto, 606-8507 Japan; 4 Department of Human Health Sciences, Graduate School of Medicine, Kyoto University, Kyoto, Japan

**Correction to: International Journal of Hematology (2026) 123:421–431** 10.1007/s12185-025-04109-7.

In Table 3 of this article, numerical inconsistencies were identified. Accordingly, the corrected version of Table 3 is provided in this correction.

**Table 3:** Frequency of CRS by grade for each CAR-T product

CRS grade	Total	Tisa-cel	Liso-cel	Axi-cel
	(N = 106)	(n = 76)	(n = 22)	(n = 8)
Grade 0 (No CRS)	13 (12%)	11 (14%)	2 (9%)	0 (0%)
Any CRS (Grade ≥ 1)	93 (88%)	65 (86%)	20 (90%)	8 (100%)
Grade ≥ 2 CRS	**28 (26%)**	22 (29%)	1 (5%)	**5 (63%)**
Grade ≥ 3 CRS	6 (6%)	5 (7%)	0 (0%)	1 (13%)
Grade 1a	27 (25%)	16 (21%)	9 (41%)	2 **(25%)**
Grade 1b	**38 (36%)**	27 (36%)	10 (45%)	**1 (13%)**
Grade 2	**22 (21%)**	17 (22%)	1 (5%)	**4 (50%)**
Grade 3	2 (2%)	2 (3%)	0 (0%)	0 (0%)
Grade 4	4 (4%)	3 (4%)	0 (0%)	1 (13%)

Abbreviations are shown in Table 1

In the sentence beginning ‘For Grade ≥ 2 CRS, the incidence…’ in this article, the number of axi-cel patients with Grade ≥ 2 CRS ‘50%’ should have read ‘63%’.

In the sentence beginning ‘In this study, Grade ≥ 2 CRS occurred…’ in this article, the number of axi-cel patients with Grade ≥ 2 CRS ‘50%’ should have read ‘63%’.

