# Supplementary material for: Low reticulocyte count at infusion is a risk factor for high-grade cytokine release syndrome in chimeric antigen receptor T cell therapy
Source: Int J Hematol. 2025 Nov 17;123(3):421–31. doi: 10.1007/s12185-025-04109-7 (PMC12967528; doi:10.1007/s12185-025-04109-7)
Supplement: Supplementary file 1 — Supplementary file1 (PDF 292 KB) [file 12185_2025_4109_MOESM1_ESM.pdf]

Supplementary Information

Low Reticulocyte Count at Infusion is a Risk Factor for High-Grade Cytokine Release Syndrome in Chimeric Antigen Receptor T Cell Therapy

Yusuke Tashiro, Tomoyasu Jo, Toshio Kitawaki, Noriyoshi Yoshinaga, Takashi Sakamoto, Kotaro Shirakawa, Junya Kanda, Momoko Nishikori, Kouhei Yamashita, Miki Nagao, Akifumi Takaori-Kondo, and Yasuyuki Arai

Supplemental Table 1. Univariate Analysis for Grade ≥2 CRS

	HR(95% CI)	p-value
**Gender**		
Female	1	Reference
Male	1.26 (0.62–2.57)	0.52
**Age at infusion (per year)**	1.02 (0.98–1.05)	0.37
**Disease**		
FL	1	Reference
DLBCL	1.34 (0.18–9.90)	0.77
**Transformed (in DLBCL)**		
No	1	Reference
Yes	1.11 (0.49–2.51)	0.80
**Hans (in DLBCL)**		
non-GCB	1	Reference
GCB	1.24 (0.59–2.61)	0.57
**History of CNS involvement**		
**No**	1	Reference
Yes	2.50 (0.90–6.92)	0.08
**Prior lines at infusion (per line)**	0.86 (0.63–1.18)	0.36
**Disease status at infusion**		
CR/PR	1	Reference
SD/PD	1.80 (0.88–3.69)	0.11
**IPI at infusion (in DLBCL)**		
Low	1	Reference
Intermediate	1.20 (0.40–3.63)	0.75
High	2.44 (1.09–5.47)	**0.03***
**MTV at infusion (divided by median)**		
Low	1	Reference
High	2.60 (1.11–6.11)	**0.03***
**CAR-T**		
tisa-cel or liso-cell	1	Reference
axi-cell	2.92 (1.33–6.43)	**<0.01***
**ICANS**		
No	1	Reference
Yes	4.76 (2.34–9.70)	**<0.01***
**Best response**		
CR/PR	1	Reference
SD/PD	1.22 (0.50–2.99)	0.67
**Day 0**		
Reticulotyte count (per log)	0.14 (0.05–0.46)	**<0.01***
WBC count (per log)	0.25 (0.11–0.57)	**<0.01***
Neut (%) (per %)	0.99 (0.97–1.02)	0.52
Lymph (%) (per %)	0.99 (0.96–1.02)	0.55
Mono (%) (per %)	1.01 (0.93–1.09)	0.84
Eosino (%) (per %)	1.00 (0.95–1.05)	0.95
Baso (%) (per %)	0.90 (0.56–1.47)	0.68
LDH (U/L) (per log)	8.61 (1.32–56.41)	**0.03***
CRP (mg/dL) (per log)	2.27 (1.23–4.19)	**<0.01***
Plt ($\times 10^4$ /μL) (per 10^4 /μL)	0.98 (0.92–1.03)	0.39
mEASIX score (per log)	1.81 (1.18–2.76)	**<0.01***

Abbreviations: axi-cel, axicabtagene ciloleucel; baso, basophils; CAR-T, chimeric antigen receptor-T cell; CI, confidence interval; CNS, central nervous system; CR, complete response; CRS, cytokine-release syndrome; DLBCL, diffuse large B-cell lymphoma; eosino, eosinophils; FL, follicular lymphoma; GCB, germinal center B-cell–like; HR, hazard ratio; ICANS, immune effector cell-associated neurotoxicity syndrome; IPI, international prognostic index; liso-cel, lisocabtagene maraleucel; lymph, lymphocytes; mEASIX, modified Endothelial Activation and Stress Index; mono, monocytes; MTV, metabolic tumor volume; neut, neutrophils; PD, progressive disease; plt, platelet; PR, partial response; SD, stable disease; tisa-cel, tisagenlecleucel; WBC, white blood cell. * indicates p<0.05.

Supplemental Table 2. Relationship Between CRS Grade and Reticulocyte Count

CRS Grade	**Total** (N=104)	**Low Reticulocytes** (n=28)	**High Reticulocytes** (n=76)	p-value
Grade 0 (No CRS)	12 (12%)	3 (11%)	9 (12%)	
Grade 1a	27 (26%)	6 (21%)	21 (28%)	
Grade 1b	38 (37%)	7 (25%)	31 (41%)	
Grade 2	21 (20%)	7 (25%)	14 (18%)	
Grade 3	2 (2%)	2 (7%)	0 (0%)	
Grade 4	4 (4%)	3 (11%)	1 (1%)	
Any CRS	92 (88%)	25 (89%)	67 (88%)	1
CRS (Grade≥2)	27 (26%)	12 (43%)	15 (20%)	**0.024***
CRS (Grade≥3)	6 (6%)	5 (18%)	1 (1%)	**0.005***

Abbreviations: CRS, cytokine release syndrome. * indicates p<0.05.

Supplemental Table 3. Scoring of the Fine-Gray Hazard Model

	Log HR (β)	Point
Reticulocyte < 15,000/μL	0.793	1
MTV ≥ 100mL	0.967	1
Use of axi-cel	0.971	1

Abbreviations are shown in Supplemental Table 1.

Supplemental Table 4. Relationship Between CRS Grade and KyoTox-CRS

CRS Grade	Total (N=93)	0 points (n=49)	1 point (n=30)	2 points (n=13)	3 points (n=1)	p-value
Grade 0 (No CRS)	11 (12%)	7 (14%)	3 (10%)	1 (8%)	0 (0%)	
Grade 1a	24 (26%)	15 (31%)	7 (23%)	2 (15%)	0 (0%)	
Grade 1b	35 (38%)	21 (43%)	11 (37%)	3 (23%)	0 (0%)	
Grade 2	18 (19%)	5 (10%)	9 (30%)	4 (31%)	0 (0%)	
Grade 3	1 (1%)	0 (0%)	0 (0%)	1 (8%)	0 (0%)	
Grade 4	4 (4%)	1 (2%)	0 (0%)	2 (15%)	1 (100%)	
Any CRS	82 (88%)	42 (86%)	27 (90%)	12 (92%)	1 (100%)	0.918
CRS (Grade≥2)	23 (25%)	6 (12%)	9 (30%)	7 (54%)	1 (100%)	**0.002***
CRS (Grade≥3)	5 (5%)	1 (2%)	0 (0%)	3 (23%)	1 (100%)	**<0.001***

Abbreviations: CRS, cytokine release syndrome. * indicates p<0.05.

Supplemental Figure 1

(A)

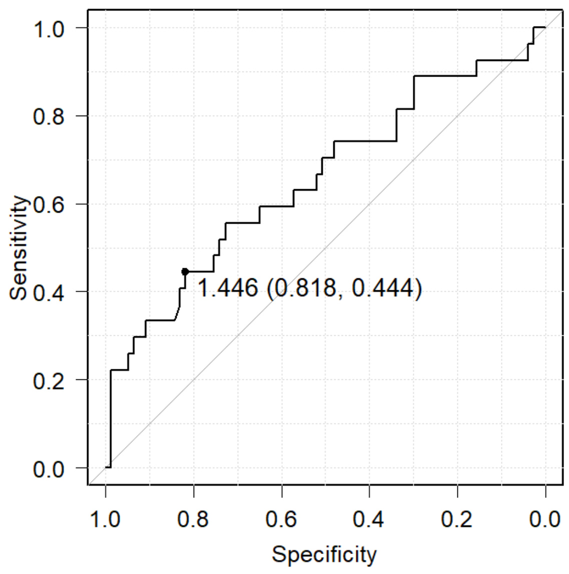

(B)

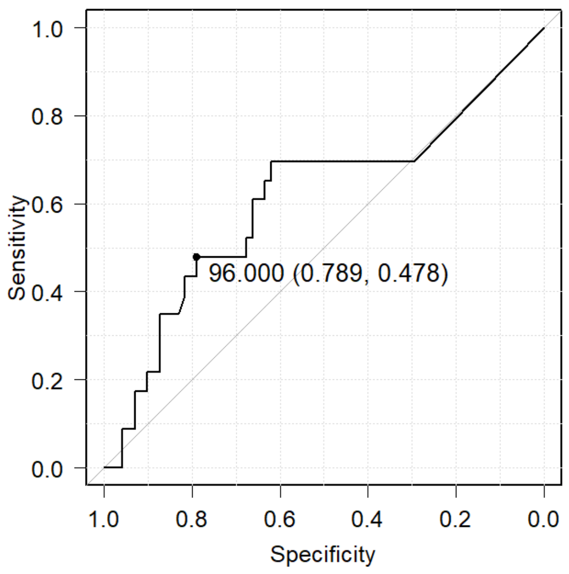

(C)

	HR (95% CI)	p-value
Reticulocyte at infusion		
≥ 15,000/μL	1	Reference
< 15,000/μL	2.55 (1.23–5.27)	**0.01***
MTV at infusion		
< 100mL	1	Reference
≥ 100mL	2.85 (1.30–6.23)	**<0.01***

Supplemental Figure 1. Receiver operating characteristic (ROC) curve analysis for reticulocyte count and metabolic tumor volume (MTV). (A) Reticulocyte count. **(B)** MTV. **(C)** Univariate analysis of Grade ≥2 CRS using the cutoff value of reticulocyte count and MTV. * indicates $p<0.05$.

Supplemental Figure 2

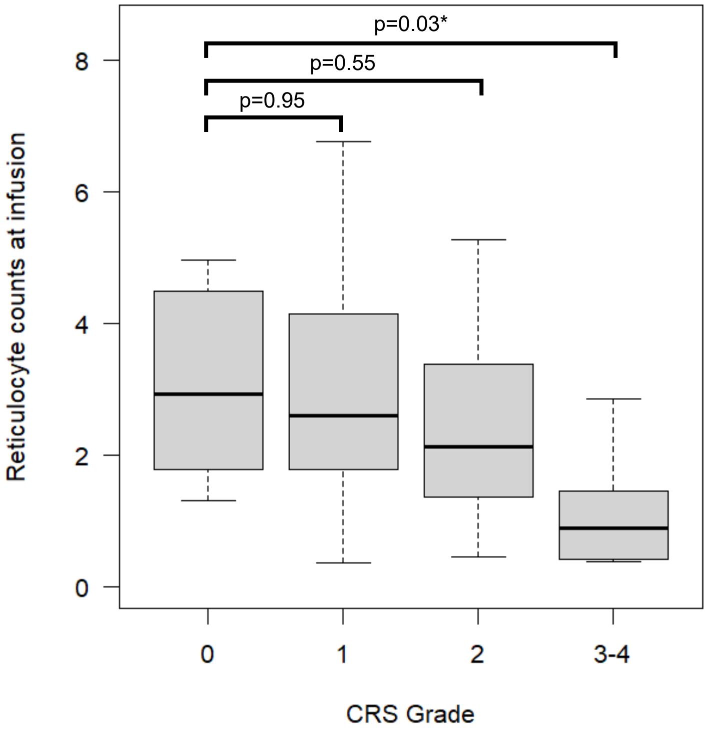

Supplemental Figure 2. Relationship between cytokine release syndrome (CRS) grade and reticulocyte count. The box plot shows reticulocyte counts across different CRS Grades. Reticulocyte counts were significantly lower in patients with higher CRS Grades (p=0.021). Group comparisons were performed by one-way ANOVA followed by Tukey's post hoc test. * indicates p<0.05.

Supplemental Figure 3

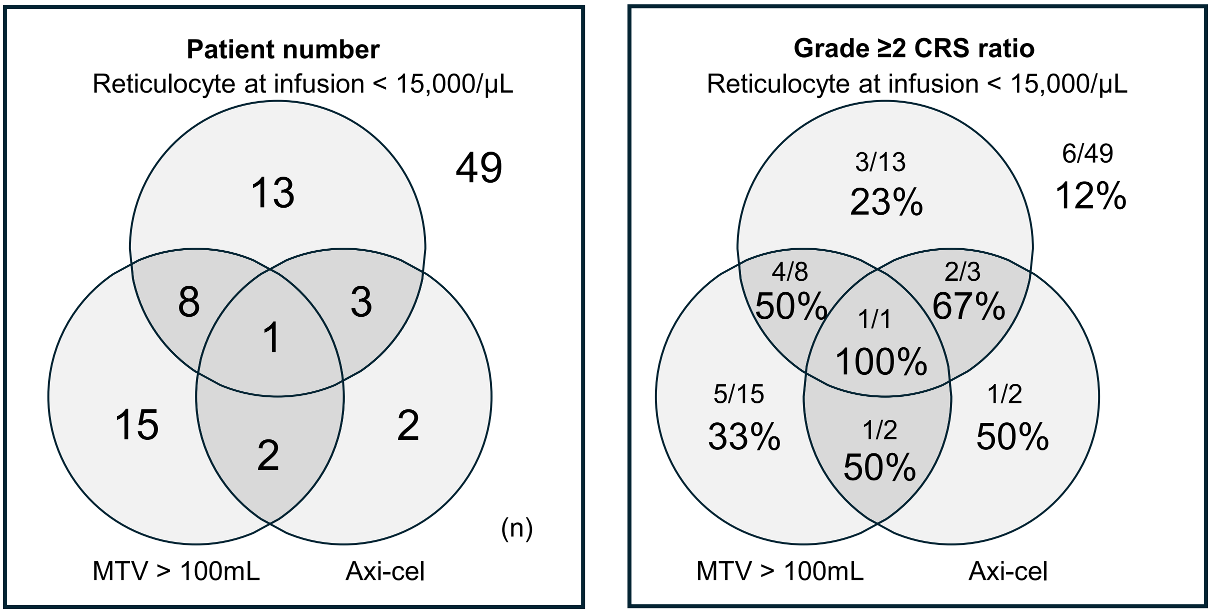

Supplemental Figure 3. Distribution of Patients According to KyoTox-CRS. Venn diagrams illustrate the overlap of the three risk factors contributing to the prediction of high-grade CRS in each risk group. Areas shaded in light gray indicate an intermediate risk, whereas those shaded in dark gray indicate a high risk. The numbers and percentages of patients exhibiting each factor are indicated as follows: Intermediate-risk group: low reticulocyte count (n = 13, 43%), high MTV (n = 15, 50%), axi-cel use (n = 2, 7%). High-risk group: low reticulocyte count (n = 12, 86%), high MTV (n = 11, 79%), axi-cel use (n = 6, 43%). Abbreviations are shown in Supplemental Table 1.
